# A cross-sectional study on attitudes toward gender equality, sexual behavior, positive sexual experiences, and communication about sex among sexually active and non-sexually active adolescents in Bolivia and Ecuador

**DOI:** 10.3402/gha.v7.24089

**Published:** 2014-07-11

**Authors:** Sara De Meyer, Lina Jaruseviciene, Apolinaras Zaborskis, Peter Decat, Bernardo Vega, Kathya Cordova, Marleen Temmerman, Olivier Degomme, Kristien Michielsen

**Affiliations:** 1 International Centre for Reproductive Health (ICRH), Ghent University, Ghent, Belgium; 2 Department of Family Medicine, Lithuanian University of Health Sciences (LUHS), Kaunas, Lithuania; 3 Faculty of Medicine, University of Cuenca, Cuenca, Ecuador; 4 South Group, Cochabamba, Bolivia

**Keywords:** adolescents, gender attitudes, Latin America, sexual behavior, positive sexual experiences

## Abstract

**Background:**

It is widely agreed upon that gender is a key aspect of sexuality however, questions remain on how gender exactly influences adolescents’ sexual health.

**Objective:**

The aim of this research was to study correlations between gender equality attitudes and sexual behavior, sexual experiences and communication about sex among sexually active and non-sexually active adolescents in 2 Latin American countries.

**Design:**

In 2011, a cross-sectional study was carried out among 5,913 adolescents aged 14–18 in 20 secondary schools in Cochabamba (Bolivia) and 6 secondary schools in Cuenca (Ecuador). Models were built using logistic regressions to assess the predictive value of attitudes toward gender equality on adolescents’ sexual behavior, on experiences and on communication.

**Results:**

The analysis shows that sexually active adolescents who consider gender equality as important report higher current use of contraceptives within the couple. They are more likely to describe their last sexual intercourse as a positive experience and consider it easier to talk with their partner about sexuality than sexually experienced adolescents who are less positively inclined toward gender equality. These correlations remained consistent whether the respondent was a boy or a girl. Non-sexually active adolescents, who consider gender equality to be important, are more likely to think that sexual intercourse is a positive experience. They consider it less necessary to have sexual intercourse to maintain a relationship and find it easier to communicate with their girlfriend or boyfriend than sexually non-active adolescents who consider gender equality to be less important. Comparable results were found for boys and girls.

**Conclusions:**

Our results suggest that gender equality attitudes have a positive impact on adolescents’ sexual and reproductive health (SRH) and wellbeing. Further research is necessary to better understand the relationship between gender attitudes and specific SRH outcomes such as unwanted teenage pregnancies and sexual pleasure among adolescents worldwide.

It is widely agreed upon that gender is a key aspect of sexuality (1–4); however, questions remain on how gender exactly influences adolescents’ sexual health (2, 5, 6). The relationship between gender and sexuality is a multifaceted and multi-determined social process (7), strongly affected by societal, interpersonal, and personal factors (1, 8). During adolescence, sexual and reproductive development and health are key issues that go hand in hand with gender equality (2, 9). Studies have shown that less egalitarian gender norms threaten the sexual health and wellbeing of adolescents (2, 10–13). Adolescent boys with less egalitarian gender norms are more likely to engage in sexual risk behavior, such as having multiple sexual partners. Adolescent girls with less egalitarian gender norms are more vulnerable to negative sexual and reproductive health (SRH) outcomes, such as experiencing sexual coercion (6, 8, 14).

## Background

On a societal level, former research clearly indicated that social influences and cultural attitudes have an effect on sexual behavior among adolescents (2, 8, 14). Different gender norms exist for adolescent boys and girls (2). Adolescents internalize these social norms and values before they become sexual active (15) and their sexual attitudes and behavior are shaped by them (6, 14, 16).

On an interpersonal level, research indicated the need to further explore factors that are related to adolescent communication about sexuality (17, 18). It has been proven that gender stereotypes shape the way young people communicate (19) and that communication about sexuality is different for adolescent boys and girls (18, 20–22).

On an individual level, aspects such as norms and attitudes influence adolescents’ sexual behavior and experiences. Girls tend to have more egalitarian gender role attitudes than boys who claim to have more traditional gender role attitudes (6, 23). These differences play a role in diverse sexual behavior for both sexes (2). Little is known on the association between adolescent sexual behavior and sexual pleasure (2, 24). However, research has proven that adolescents consider sexual pleasure to be an important goal in their relationships and that they expect sex to result in sexual pleasure (13). Studies among students in the United States have shown that gender equality and perceived equity in a relationship are both associated with sexual enjoyment (12, 25). Similar data were found among Swedish adolescent heterosexual girls who mentioned sexual pleasure on equal terms as a characteristic of their ideal sexual situation (26).

Limited research in Latin America confirmed these results. At societal level, Latino cultures are characterized by a cultural machismo–marianismo system, which includes a traditional gender ideal of male dominance and female submission (10, 27, 28). Studies in the Caribbean and Ecuador indicated that these diverse social and cultural gender norms lead to different sexual behavior among boys and girls (8, 28). The macho male adolescents are supposed to be heterosexual, have many sexual partners and should engage in higher sexual risk behavior than the female adolescents who are expected to be innocent and self-sacrificing and therefore more vulnerable to negative SRH outcomes (8, 28, 29). These traditional gender norms also constitute barriers for adolescent girls to enjoy sexual experiences (10, 28).

At an interpersonal level, research in the United States has shown that a cultural Latin American background increases the difficulty to communicate on sexuality (30, 31). The Horizon project in Brazil concluded that boys who participated in interventions that promoted gender equitable behavior, communicated with their primary partners about a broader range of key HIV/STI-related topics (29).

At an individual level, the same project indicated that men who had more equitable gender norms showed less sexual risk behavior (32). Research in Ecuador and Brazil found an increasing tendency among adolescents to have more consensual and pleasant sex and depicts a close relationship between gender norms and adolescent sexual pleasure (10, 28, 32).

We can conclude that evidence on the association between gender and adolescent sexuality exists and is growing. The importance of gender for adolescents’ sexuality is also recognized by international organizations such as the United Nations Population Fund and the World Health Organization (WHO) who recognize the need to address gender as an ‘upstream’ antecedent of adolescents’ sexual health behavior (33, 34). However, until now in Latin America, only limited research was conducted on societal, interpersonal and personal levels to understand the link between gender equality and adolescents’ sexuality (8, 10, 27–29, 32). On a societal level, comparing different gender equality indicators could assess this relationship. Nevertheless, our research focusses on the individual and interpersonal level, while taking into account that adolescents remain the main target group in changing behavior programs to improve their sexual health. We defined the interpersonal level as the level which includes factors that are related to the interaction of the adolescent with their partners, peers and parents. The main objective of this article is to describe how gender equality attitudes among adolescents in Latin America are correlated to their sexual behavior, positive sexual experiences and communication about sex. To the best of our knowledge, these correlations have not yet been structurally studied in any large-scale research performed in Latin America.

## Methods

This paper presents partial results of the international interventional research project Community Embedded Reproductive Health Care for Adolescents in Latin America (CERCA), funded by the European Commission (35). CERCA seeks to create a community-based model to improve adolescent health, by organizing activities such as workshops, family visits, sending informative text messages and psychological counseling. The topics treated were related to SRH and wellbeing for adolescents, communities, health care providers and authorities. The intervention ran for a period of approximately 2 years.

In 2011, a cross-sectional study was carried out among 5,913 adolescents aged 14–18 years in 20 secondary schools in Cochabamba (Bolivia) and 6 secondary schools in Cuenca (Ecuador). These schools were purposively selected according to a strategy developed by the CERCA consortium: 1) selection of primary health care centers that took part in the interventions: 2 in Cochabamba and 3 in Cuenca; 2) selection of secondary intervention schools that fell within the area of coverage of these health care centers: 12 in Cochabamba and 3 in Cuenca; and 3) selection of secondary control schools within the area of coverage of other primary health care centers: 8 in Cochabamba and 3 in Cuenca. The selected intervention and control schools had similar characteristics (socio-economic indicators, geographic location and the size of school). The survey was conducted in both intervention and control schools (20 out of a total of 1,100 schools in Cochabamba and 6 out of a total of 127 schools in Cuenca) before the intervention started. The amount of selected schools and participants was based on the calculations for a cross-sectional control study measuring the impact of interventions on contraceptive use among adolescents. We estimated that among 14–18 year-old adolescents, 30% are sexually active and that 30% of the sexually active adolescents use a modern contraceptive. Using the finite population correction factor, we determined that a minimum of 2,057 respondents was needed in each country to detect a significant difference of 10% in contraceptive use between the intervention and control groups. Due to the larger amount of schools in Cochabamba, within the area of coverage of the selected health care centers, more schools were selected in Cochabamba than in Cuenca. In Cochabamba, the selected health care centers and schools were located in 3 different zones, which are all urban with basic health services. One zone is mainly populated by middle-income families (Sarcobamba); the other 2 areas (Central Cochabamba and Quintanilla) have a mix of very poor and very rich inhabitants. In Cuenca, the survey was conducted in urban (parish Cañaribamba) and rural areas (parish El Valle and Chiquintad). The parish of Chiquintad is characterized by migration and poverty; the other 2 parishes have residents of middle-income. Due to the fact that the selected schools in Cuenca were mainly technical schools, girls were underrepresented in our sample. However, our sample had a representative mix of adolescents with an urban versus rural (only Ecuador) and diverse socio-economic background.

In Bolivia, the study was approved on 6 July 2011 and in Ecuador on 13 September 2011 by the relevant ethical committees (Tribunal de Ética Médica Colegio Médico de Cochabamba and Comisión de Bioética Facultad de Ciencias Médicas, Universidad de Cuenca). In both countries, different procedures were applied, depending on the national legislation. In Bolivia, the permission of the Ministry of Education and institutional permission of all selected schools was obtained. Subsequently, trained CERCA staff invited all 14–18 year-old students who were present that day, to complete a self-administered questionnaire after having signed an informed consent form (36). In the selected schools in Ecuador, firstly, institutional permission was obtained and secondly, all parents or guardians of the students were, during a school meeting, informed by project staff about the study. Afterwards, they were asked to provide written refusal or agreement on the participation of their children. The students who had parental permission to participate were asked to sign an informed consent. Of all Ecuadorian students who received approval of their parents to participate (all but 11), 2 refused to take part in the study. In Bolivia all invited adolescents participated. In total, 4,000 adolescents in Bolivia and 2,699 in Ecuador (total 6,699 adolescents), aged between 14 and 18, took part in the research. However, 486 questionnaires in Bolivia and 300 in Ecuador were invalid and excluded from the study. This brought the total final sample down to 5,913 adolescents.

One of the investigated topics was adolescents’ gender attitudes, which was measured using the Attitudes toward Women Scale for Adolescents (AWSA) (37). This scale is widely used to assess gender attitudes among adolescents (38–42). Galambos et al. found high reliability for the internal consistency estimates and the test–retest stability of the scale as well as a large support for the construct validity of the scale (37). For this study, the Spanish version of AWSA was used. The scale was pilot tested before conducting the survey.

A factor analysis of the AWSA scale was performed and is described in detail in the authors’ article on the AWSA scale (36). Three subscales emerged from the factor analysis: power dimension, equality dimension and behavioral dimension. When testing the external validity of the different factors, mainly the factor of equality dimension revealed a consistent correlation with adolescent sexual behavior. This article provides a more in-depth analysis of the equality dimension subscale. Respondents were asked if they agreed with the following 4 statements which refer to the fact that men and women should have the same rights and opportunities (in brackets the number of the item in the AWSA is presented):

(V03) On average, girls are as smart as boys
(V05) It is alright for a girl to want to play rough sports like football
(V09) If both husband and wife have jobs, the husband should do a share of the housework such as washing dishes and doing the laundry
(V12) Girls should have the same freedom as boys

Each item represents an attitude to which the study participants responded on a 4-point Likert-type scale ranging from 1 ‘strongly agree’ to 4 ‘strongly disagree’. In the analyses, the responses were reversely coded to create a gradient with higher scores indicating a more positive attitude toward gender equality. For each item, on average 40 (0.7%) values were missing. They were replaced by the estimated mean from the respondent's answers to the remaining items. For further analysis, the values of the factorial scores on the equality dimension were divided in 3 categories, using their terciles as cut-off points and coded as: 1=low, 2=medium and 3=high positive attitude toward gender equality. Due to differences in responses, this procedure was performed by splitting data into: respondents’ sex, age group (14–16 and 17–18) and country.

The Cronbach α was used as a measure of internal consistency of the subscale. The subscale was subjected to the Spearman–Brown prediction formula to adjust its reliability to the reliability of the full 12-item test (43). A Cronbach *α*≥0.70 (0.70 for girls and 0.73 for boys) was considered acceptable.

The statistical analyses were done using SPSS 21.0. As the sample of our study was not randomly selected, in all analyses, data were adjusted through weighing by sex, country and age, using the average distribution of the respondents as a standard population. By weighing, we aimed at reflecting the distribution in the general study population.

Logistic regressions were performed to estimate the adjusted odds ratios (aORs) and their 95% confidence intervals of adolescents’ sexual behavior, sexual experiences and communication about sex in relation to adolescents’ attitudes toward gender equality. The goodness of fit of the logistic regression model was evaluated calculating the Hosmer and Lemeshow test (*p*>0.05 indicating an acceptable model). Interactions between sex and gender equality attitudes (both categorical variables) were tested.

Sexual behavior was measured, using the following questions and variables: ‘Did you ever have sexual intercourse (coitus)?’ (yes/no), ‘Do or did you feel pressure to have sexual intercourse because a lot of your peers already had sexual intercourse?’ (yes/no), number of sexual partners (2 or more/1), current contraceptive use of couples (yes/no) (current use of contraceptives or the use of a condom during the 3 most recent sexual experiences), the fact whether both had taken the initiative to have sexual intercourse the last time (yes/no) and the agreement on the necessity to have sexual intercourse to maintain a relationship (yes/no).

Positive sexual experiences for sexually active adolescents were measured by the outcomes ‘positive experience’ and ‘not positive experience’ (neutral, negative, don't know) on the question ‘How did you feel the last time you had sexual intercourse?’ For sexually inactive adolescents a bivariate variable was formulated based on the question ‘Do you think that sexual intercourse is a positive experience?’

The bivariate variable (yes/no) ‘easy communication with the partner’ is based on the answers of adolescents who indicated currently having a partner.

We assessed the predictive role of attitudes toward gender equality in different components of adolescents’ sexual behavior, experiences and communication for sexually active and for sexually non-active adolescents separately. The same confounding factors (age, sex, country, living with mother/father, living conditions, and importance of religion) were included in both models as adjusting components. The confounders were identified based on correlation analysis and on literature research.

## Results

Of the 5,913 respondents, 3,330 were boys and 2,583 were girls, 59.4% were Bolivian and 40.6% Ecuadorian. Of all respondents 23.4% ever had sexual intercourse. In the overall sample, 93.9% of the respondents completed the AWSA scale. One hundred and thirty-seven respondents (2.3%) did not respond to any of the items V03, V05, V09 and V12 and were therefore excluded from the analysis. Table 1 describes the crude and the weighted distribution of respondents by social, demographic and sexual outcome variables.

**Table 1:** Crude and weighted distribution of respondents by social, demographic and sexual outcome variables

	Crude number of cases	Weighted number of cases
	
Characteristics (predictors and outcome variables)	*n* (%)	*n* (%)
**All cases**	5,913 (100.0)	5,913 (100.0)
Sex		
Boys	3,330 (56.3)	2,957 (50.0)
Girls	2,583 (43.7)	2,956 (50.0)
Age (years)		
14	1,173 (19,8)	1,183 (20.0)
15	1,451 (24.5)	1,183 (20.0)
16	1,456 (24.6)	1,183 (20.0)
17	1,274 (21.5)	1,182 (20.0)
18	559 (9.5)	1,182 (20.0)
Country		
Bolivia	3,514 (59.4)	2,957 (50.0)
Ecuador	2,399 (40.6)	2,956 (50.0)
Living with mother during the last 3 years		
Less than last 3 years	1,043 (18.2)	1,034 (18.1)
3 years or more	4,696 (81.8)	4,691 (81.9)
Living with father during the last 3 years		
Less than last 3 years	2,273 (39.5)	2,437 (42.4)
3 years or more	3,486 (60.5)	3,315 (57.6)
Quality of living house		
Poor	2,069 (35.0)	2,125 (36.0)
Good	3,834 (65.0)	3,779 (64.0)
Importance of religion		
Not important	1,487 (26.9)	1,344 (24.3)
Important	4,047 (73.1)	4,186 (75.7)
Factor of gender equality		
Low	1,963 (33.2)	1,971 (33.4)
Middle	2,077 (35.1)	2,042 (34.5)
High	1,873 (31.7)	1,900 (32.1)
Had sexual intercourse (penetration)		
No	4,518 (76.6)	4,341 (73.6)
Yes	1,379 (23.4)	1,557 (26.4)
**Those who had sex**	1,379 (100.0)	1,557 (100.0)
Number of sexual partners		
1	596 (51.2)	719 (53.6)
2 or more	569 (48.8)	622 (46.4)
Actual use of contraceptives		
No	954 (69.2)	1,054 (67.7)
Yes	425 (30.8)	502 (32.3)
Experience of last sexual intercourse		
Not positive	518 (39.5)	531 (35.8)
Positive	794 (60.5)	953 (64.2)
Mutual initiative to have sexual intercourse the last time		
No	767 (55.6)	798 (51.3)
Yes	612 (44.4)	758 (48.7)
Pressure to have sexual intercourse		
No	1,010 (85.6)	1,169 (86.9)
Yes	170 (14.4)	175 (13.1)
Easy communication with partner about sex		
No	432 (37.8)	457 (36.3)
Yes	712 (62.2)	802 (63.7)
**Those who did not have sex**	4,518 (100.0)	4,341 (100.0)
Ideas about sexual intercourse		
Not positive	3,336 (76.8)	3,245 (77.5)
Positive	1,005 (23.2)	943 (22.5)
Agreement with necessity to have sexual intercourse to maintain a relationship		
Did not agree	3,271 (74.4)	3,205 (75.7)
Agreed or did not know	1,128 (25.6)	1,031 (24.3)
Feeling the pressure to have sexual intercourse		
No	2,991 (89.3)	2,884 (89.5)
Yes	359 (10.7)	340 (10.5)
Easy communication with partner about sex		
No	985 (69.3)	952 (68.5)
Yes	436 (30.7)	437 (31.5)

Table 2 displays the distribution of the scores per AWSA item by sex and age.

**Table 2:** The distribution of the scores per AWSA item by sex and age

	Distribution of responses to the items of the gender equality subscale^a^							
	
	V03		V05		V09		V12	
				
Group of respondents	Disagree	Agree	Disagree	Agree	Disagree	Agree	Disagree	Agree
Boys	770 (26.3%)	2,157 (73.7%)	1,041 (35.3%)	1,904 (64.7%)	260 (8.8%)	2,679 (91.2%)	667 (22.7%)	2,271 (77.3%)
Girls	508 (17.3%)	2,426 (82.7%)	808 (27.6%)	2,120 (72.4%)	127 (4.3%)	2,805 (95.7%)	455 (15.5%)	2,486 (84.5%)
*p*^b^	<0.001		<0.001		<0.001		<0.001	
14–16-year old adolescents	808 (23.0%)	2,707 (77.0%)	1,158 (32.9%)	2,363 (67.1%)	264 (7.5%)	3,259 (92.5%)	610 (17.3%)	2,911 (82.7%)
17–18-year old adolescents	469 (20.0%)	1,876 (80.0%)	692 (29.4%)	1,661 (70.6%)	123 (5.2%)	2,225 (94.8%)	512 (21.7%)	1,846 (78.3%)
*p*^b^	0.007		0.005		0.001		<0.001	

a Responses ‘Strongly disagree’ and ‘Disagree’ were aggregated to ‘Disagree’ and responses ‘Agree’ and ‘Strongly agree’ were aggregated to ‘Agree’;

b Chi-square test.

The mean of the total score on the gender equality subscale was 12.68 and the median 13.00. Girls expressed more positive attitudes toward gender equality than boys (mean scores were 13.10 and 12.27, *p*<0.001, respectively for girls and boys). The scores did not significantly differ by age or by whether or not the adolescents were sexually active.

Table 3 shows the results of the logistic regression for sexually active adolescents. Adolescents who were sexually active and who considered gender equality as important (high vs. low) declared higher current use of contraceptives within the couple, were more likely to describe their last sexual intercourse as a positive experience and considered it easier to communicate with their partner about sex than sexually experienced adolescents who were less positively inclined toward gender equality. Gender equality attitudes were not a significant predictor of ever having had sexual intercourse, of the number of sexual partners, of mutual initiative to have sexual intercourse, or of pressure for sexual intercourse. When calculating the interaction between sex and gender equality, the outcome remained consistent.

**Table 3:** Demographic and social factors predicting different aspects of sexual behavior among sexually active adolescents: odds ratios (ORs) and 95% confidence intervals (CIs) estimated from the multivariate binary logistic regression

	Ever had sexual intercourse		Number of sexual partners		Current use of contraceptives		Positive experience last sexual intercourse		Both have taken initiative to have sexual intercourse the last time		Pressure for sexual intercourse		Easy communication with partner	
							
Predictors	OR	(95% CI)	OR	(95% CI)	OR	(95% CI)	OR	(95% CI)	OR	(95% CI)	OR	(95% CI)	OR	(95% CI)
Age^a^	**1.85**	(1.75; 1.95)***	**1.17**	(1.06; 1.30)**	**1.17**	(1.06; 1.29)**	**1.20**	(1.09; 1.33)***	**1.31**	(1.20; 1.44)***	0.92	(0.80; 1.06)	**1.18**	(1.06; 1.31)**
Country^b^	**1.21**	(1.05; 1.39)**	0.98	(0.76; 1.26)	**1.33**	(1.05; 1.70)*	**1.81**	(1.42; 2.31)***	**1.51**	(1.23; 1.94)***	**0.68**	(0.47; 0.97)*	1.15	(0.88; 1.51)
Sex^c^	**2.17**	(1.89; 2.50)***	**3.1**	(2.41; 3.98)***	0.88	(0.69 1.18)	0.79	(0.62; 1.02)	**0.65**	(0.52; 0.82)***	0.85	(0.60; 1.22)	0.77	(0.58; 1.01)
Gender equality^d^	0.9	(0.77; 1.07)	1.01	(0.75; 1.36)	**1.52**	(1.14; 2.03)**	1.13	(0.85; 1.50)	1.27	(0.97; 1.66)	0.89	(0.57; 1.38)	1.06	(0.79; 1.44)
Gender equality^e^	0.98	(0.84; 1.16)	1.16	(0.87; 1.55)	**1.67**	(1.26; 2.21)***	**1.71**	(1.28; 2.28)***	1.21	(0.93; 1.58)	1.28	(0.85; 1.92)	**1.83**	(1.34; 2.50)**
Living with mother^f^	**0.61**	(0.51; 0.72)***	**0.72**	(0.53; 0.97)*	0.83	(0.62; 1.10)	0.88	(0.65; 1.18)	1.10	(0.83; 1.45)	**0.42**	(0.28; 0.62)***	0.88	(0.64; 1.23)
Living with father^f^	**0.77**	(0.66; 0.89)***	0.95	(0.73; 1.23)	1.15	(0.89; 1.48)	**1.44**	(1.11; 1.86)**	0.90	(0.70; 1.14)	1.12	(0.76; 1.63)	**0.75**	(0.57; 0.99)*
Living conditions^g^	**1.16**	(1.01; 1.34)*	**1.37**	(1.05; 1.78)*	1.13	(0.88: 1.45)	**1.34**	(1.04; 1.73)*	1.12	(0.88; 1.42)	0.80	(0.55; 1.16)	**2.05**	(1.56; 2.69)***
Importance of religion^h^	**0.61**	(0.53; 0.72)***	**0.6**	(0.45; 0.79)***	0.87	(0.67; 1.13)	**1.64**	(1.27; 2.12)***	**1.32**	(1.03; 1.69)*	**0.69**	(0.48; 1.00)*	**0.68**	(0.51; 0.92)*
*p*^i^	0.853		0.133		0.098		0.090		0.090		0.058		0.155	

a Change by 1 year;

b Ecuador vs. Bolivia (ref.);

c boys vs. girls (ref.);

d medium vs. low gender equality (ref.);

e high vs. low gender equality (ref.);

f during last 3 years, lived together with father/mother all time vs. not all time (ref.);

g good vs. poor (ref.);

h religion was considered as a value vs. not a value (ref);

i Hosmer and Lemashow test to evaluate the goodness of fit of the logistic regression model.

* *p*<0.05;

** *p*<0.01;

*** *p*<0.001 (bolded).

The same analysis was done for non-sexually active adolescents (Table 4). In this group, adolescents who considered gender equality as important, were more likely to think that sexual intercourse is a positive experience, considered it less necessary to have sexual intercourse to maintain a relationship and found it easier to communicate about sex with their girlfriend or boyfriend than sexually non-active adolescents who were less supportive toward gender equality. An association between gender equality attitudes and pressure to have sexual intercourse has not been found. Similar aOR have been obtained when including the interaction between sex and gender equality in the model.

**Table 4:** Demographic and social factors predicting different aspects of sexual behavior among adolescents who haven't had sexual intercourse yet: odds ratios (ORs) and 95% confidence intervals (CIs) estimated from the multivariate binary logistic regression

	Positive ideas about sexual intercourse		Agreement to have sexual intercourse to maintain a relationship		Easy communication with partner about sex		Feeling pressure to have sexual intercourse	
				
Predictors	OR	(95% CI)	OR	(95% CI)	OR	(95% CI)	OR	(95% CI)
Age^a^	**1.17**	(1.10 1.24)***	**1.09**	(1.03; 1.15)**	**1.30**	(1.21; 1.40)***	**1.20**	(1.10; 1.31)***
Country^b^	**1.57**	(1.33; 1.86)***	0.96	(0.82; 1.13)	**0.81**	(0.66; 1.00)*	**0.73**	(0.57; 0.93)*
Sex^c^	**3.75**	(3.16; 4.44)***	**3.67**	(3.11; 4.32)***	**1.64**	(1.34; 2.02)***	**2.22**	(1.74; 2.84)***
Gender equality^d^	**1.30**	(1.06; 1.59)*	**0.79**	(0.65; 0.95)***	**1.41**	(1.10; 1.81)**	0.94	(0.70; 1.25)
Gender equality^e^	**1.36**	(1.11; 1.66)**	**0.60**	(0.49; 0.73)***	**1.86**	(1.44; 2.39)***	0.93	(0.70; 1.24)
Living with mother^f^	**1.32**	(1.03; 1.71)*	1.11	(0.87; 1.41)*	0.89	(0.67; 1.18)	0.87	(0.65; 1.17)
Living with father^f^	0.91	(0.77; 1.09)	1.09	(0.92; 1.30)	1.03	(0.83; 1.28)	0.94	(0.66; 1.32)
Living conditions^g^	**1.59**	(1.34; 1.90)***	0.90	(0.77; 1.06)	1.08	(0.87; 1.33)	0.90	(0.69; 1.16)
Importance of religion^h^	1.14	(0.93; 1.39)	**0.77**	(0.64; 0.92)**	1.13	(0.86; 1.44)	0.83	(0.63; 1.10)
*p*^i^	0.173		0.223		0.716		0.234	

a Change by 1 year;

b Ecuador vs. Bolivia (ref.);

c boys vs. girls (ref.);

d medium vs. low gender equality (ref.);

e high vs. low gender equality (ref.);

f during last 3 years, lived together with father/mother all time vs. not all time (ref.);

g good vs. poor (ref.);

h religion was considered as a value vs. not a value (ref);

i Hosmer and Lemashow test to evaluate the goodness of fit of the logistic regression model.

* *p*<0.05;

** *p*<0.01;

*** *p*<0.001 (bolded).

The differences between the groups with medium and high attitudes toward gender equality are small and not significant.

Considering the confounding factors, we can observe that especially religion was positively correlated with the sexual experiences of adolescents and the mutual initiative to have the most recent sexual experience. Except for the outcome of feeling pressure to have sexual intercourse, age was also positively correlated with adolescents’ sexual behavior, experiences, and communication. Negative correlations were found between sexual active adolescents’ religion and the fact that they ever had sexual intercourse, the communication with their partner about sex, their number of sexual partners, and with feeling pressure for sexual intercourse. For non-sexually active adolescents, religion was merely negatively correlated with the agreement of needing to have sexual intercourse to be able to maintain a relationship. For these adolescents who did not have sexual intercourse yet, age is an important confounding factor for all outcomes.

## Discussion

This study investigated how gender equality attitudes among adolescents in Bolivia and Ecuador are linked with sexual topics at the individual level (mainly sexual behavior and positive sexual experiences) and at the interpersonal level (communication with partner about sex). Our study revealed that more egalitarian gender attitudes are related to higher current use of contraceptives within the couple, with more positive experiences and ideas related to sexual intercourse and easier communication about sex with the partner among sexually active and sexually non-active adolescents.

The finding of higher current contraceptive use within couples corresponds with research results found in Brazil, where intervention research indicated the link between gender equitable norms of young men and a higher reported condom use at last sexual intercourse (32). The fact that individual positive attitudes toward gender equality are related to a higher use of contraceptives is not surprising within a Latin American culture, known for its machismo. Having positive attitudes toward gender equality means one breaks free from the typical male role as virile, promiscuous and dominant and from the female stereotype as being innocent, submissive and self-sacrificing (2, 8, 14, 28). This might – at the interpersonal level – open opportunities to discuss not only topics related to HIV, as was demonstrated in Brazil (29), but also to discuss topics concerning contraceptive use. This assumption is in line with our research, which indicates a positive correlation between gender equality attitudes and communication with the partner about sexuality.

Former research indicated an association between physically measured sexual enjoyment and perceived equity among young adults in the United States (12). The research of Goicolea et al. (10) revealed an emerging interest in women's sexual pleasure among Ecuadorian adolescents. Our research is the first to indicate the relationship between attitudes in favor of gender equality and more positive experiences and ideas about sexual intercourse in Latin America.

The fact that no significant difference was found between the groups with medium and high positive attitudes toward gender equality could be related to the characteristics of the Latin American culture, known for its’ distinct gender roles for men and women. As gender equality is not yet widely accepted, the fact that adolescents’ have positive attitudes toward gender equality or not could be more important and significant then the magnitude of these attitudes. We hypothesize that the intensity of gender equality attitudes is of more importance in cultures where gender equality is well accepted.

Our data did not show a correlation of positive attitudes toward gender equality with the number of sexual partners, or with a mutual initiative to engage in the most recent sexual experience or with feeling pressure to have sexual intercourse. Former research conducted in 37 countries concluded that individuals living in highly egalitarian countries are more likely to have more sexual partners, compared to someone living in a country where women's status is significantly inferior to the status of men (44). It might be that we could not find a correlation between adolescents’ individual gender attitudes and their number of sexual partners due to the impact of gender at societal level. Bolivia and Ecuador are respectively ranked 97 and 83 in the list of the Gender Inequality Index (45). This influence of social gender norms can also explain why we did not find a relation between a mutual initiative to engage in the most recent sexual experience and gender attitudes. Regarding sexual pleasure of adolescents girls in Ecuador, Goicolea mentions that, on the individual level adolescent girls may feel equal to boys, but due to powerful cultural expectations, at the interpersonal level they may consider it inappropriate or impossible to take initiative for having sex and thus for seeking sexual pleasure (10). And finally, the fact that our study neither shows correlations between gender equality attitudes and feeling pressure to have sexual intercourse among both sexually active and non-sexually active adolescents, could be influenced by the fact that our question concerning pressure did not exclusively refer to the partner – presumed mostly of the opposite sex. The answers to our question could also imply pressure felt by peers or siblings – of both sexes. If, for example, girls reported about pressure felt by other girls, their decision to initiate sex could mainly be influenced by peer pressure and not by the gender stereotype that, being a girl, they should fulfill their boyfriend's wishes.

Our research indicated that adolescents who considered religion as important, were less likely to have developed (an extensive) sexual life. However, when they were sexually active, they demonstrated more positive experiences and mutual initiative to have sexual intercourse. This significant relationship of religiosity is of interest as it is, besides gender attitudes, an important cultural factor influencing adolescents’ sexual health and wellbeing.

Sexuality education for adolescents in Latin America is rarely widely embedded in the cultural context of a country (46) and still needs improvement. However, our research is in line with former research, which demonstrates the importance of incorporating a gender transformative approach and of promoting gender-equitable relationships between men and women to produce effective behavior that improves SRH (5). The impact of such educational programs could be measured using the ‘gender equality’ scale, which we obtained through factor analysis on the AWSA scale (36). Additionally, we would like to point out that our results suggest that these gender programs could be important for boys and for girls. Although until now, principally boys are targeted in established gender transformative projects to reduce sexual risk behavior and to prevent violence (29), our study depicts that gender attitudes are related to sexual behavior, experiences and communication of both sexes. Furthermore, our results show a correlation between positive attitudes toward gender equality and communication and ideas about sexual intercourse of adolescents who didn't have sexual intercourse yet. This implies that gender transformative programs could also be important for the sexual health and wellbeing of adolescents who are at earlier stages of their sexual trajectory (3).

Our study has various limitations. The first is related to the sampling methodology. It is important to note that the adolescents who participated in the study were not randomly chosen and they all attended schools in the city of Cochabamba (Bolivia) and Cuenca (Ecuador). Hence, we did not capture answers of adolescents in other cities nor from more vulnerable adolescents who lived in rural and poorer areas or who did not go to school. Taking into account the fact that lower socio-economic status is associated with risky sexual behavior (8) and the knowledge that education influences the gender role attitudes of adolescents (23, 47), we could expect to find different results among adolescents in broader target groups. Secondly, we are confronted with blindness within the sample, as we did not gather data on the sexual diversity of our participants. Nevertheless, in a Latin culture where many people define gender roles based on a binary biological division (man vs. woman), a relationship between sexual identity, gender attitudes and sexual behavior could be expected. Thirdly, at the level of analysis and due to the fact that our research was a sub-study within a broader investigation about SRH of adolescents, we were bound by limited socio-demographic features as confounding factors. And finally, inherent to a cross sectional study, our research did not allow to formulate causal relationships between adolescents’ gender equality attitudes and aspects related to their sexual behavior, experiences and communication with their partner.

These limitations of our study indicate the need for additional research to understand how gender has an impact on the sexual behavior of a more diverse group of adolescents. Considering the necessity to incorporate gender into a socio-ecological model of adolescent sexual health, as indicated by Pilgrim et al. (2012) and Tolman et al. (2003), we consider it important to conduct longitudinal research among a randomly selected adolescent population aiming at understanding how gender barriers function at the different levels of the socio-ecological model and how they can be removed in order to ensure healthy and satisfactory sexual health outcomes for all adolescents. Additionally, we would like to suggest to do research on comprehensive indicators for adolescent sexual pleasure. Our research results on the topic can only be viewed as a first step in the systematic measurement of sexual pleasure in Latin America.

In spite of the mentioned limitations, we were able to conduct one of the first systematically performed descriptive researches on the relationship between gender attitudes and sexual behavior, experiences and communication among a large sample of Latin American adolescents.

## Conclusion

Descriptive research in Bolivia and Ecuador has indicated a positive relationship between attitudes toward gender equality and sexual behavior, sexual experiences and communication of sexually active and non-sexually active adolescent boys and girls. Our results suggest that gender equality attitudes have a positive impact on adolescents’ SRH and wellbeing. Further research is necessary to better understand the relationship between gender attitudes and specific SRH outcomes such as unwanted teenage pregnancies and sexual pleasure among adolescents worldwide.
